# Indole-2-carboxylic Acid Nanoemulsions: A Novel Approach for Topical Cosmeceuticals with Antioxidant Properties in Human Skin Models

**DOI:** 10.1021/acsomega.6c02156

**Published:** 2026-07-31

**Authors:** Hyanne Pereira Lima, Eduarda Pires Costa, Gislaine Gonçalves Fonseca, Nathan Caraciole Brito, Manoela Maciel dos Santos Dias, Jemmyson Romário de Jesus, Tatianny de Araújo Andrade, Gaspar Diaz Muñoz, Carlos Eduardo Soares Gazzinelli Cruz, Reggiani Vilela Gonçalves, Marisa Alves Nogueira Diaz

**Affiliations:** † Department of Biochemistry and Molecular Biology, 28120 Federal University of Viçosa, Avenida PH Rolfs, s/n, Campus Universitário, Viçosa, Minas Gerais 36570-900, Brazil; ‡ Department of General Biology, 28120 Federal University of Viçosa, Avenida PH Rolfs, s/n, Campus Universitário, Viçosa, Minas Gerais 36570-900, Brazil; § Department of Chemistry, Federal University of Viçosa, Avenida PH Rolfs, s/n, Campus Universitário, Viçosa, Minas Gerais 36570-900, Brazil; ∥ Department of Animal Biology, Federal University of Viçosa, Avenida PH Rolfs, s/n, Campus Universitário, Viçosa, Minas Gerais 36570-900, Brazil; ⊥ Department of Chemistry, Universidade Federal de Minas Gerais, Belo Horizonte, Minas Gerais 31270-901, Brazil; # Department of Medicine and Nursing, Federal University of Viçosa, Avenida PH Rolfs, s/n, Campus Universitário, Viçosa, Minas Gerais 36570-900, Brazil; ∇ Department of Animal Science, Plants for Human Health Institute, North Carolina State University, Kannapolis, North Carolina 28081, United States

## Abstract

Bioactive molecules, such as indole alkaloids, have received special attention due to their remarkable pharmacological versatility, particularly their antioxidant activities. Therefore, our objective was to build an engineered delivery system (a topical nanoemulsion), incorporating indole-2-carboxylic acid, and evaluate its antioxidant potential using an *ex vivo* human skin model. The nanoemulsion was characterized by particle size, polydispersity index, zeta potential, encapsulation efficiency, *in vitro* release kinetics, and skin permeation profiles. Antioxidant enzyme activities (superoxide dismutase , catalase, glutathione S-transferase) and oxidative stress markers (carbonyl proteins, malondialdehyde) were quantified. Histological and immunohistochemical analyses assessed skin tissue responses, focusing on the oxidative stress markers [8-hydroxy-2’-deoxyguanosine (8OHdG), and 4-hydroxynonenal (4HNE)] and structural tissue markers (type III collagen, and filaggrin). Our results showed that skin permeation studies using human skin explants demonstrated effective barrier traversal, with substantial retention in the stratum corneum and recovery in the receptor compartment, supporting the formulation’s potential for transdermal delivery. The nanoemulsion exhibited no cytotoxicity in RAW 264.7 macrophages and attenuated oxidative stress, as evidenced by reduced 4HNE labeling, while enhancing key structural skin markers, including filaggrin, and preserving dermal vascularization, indicating a robust skin recovery response. In conclusion, we observed that the indole-2-carboxylic acid nanoemulsion effectively modulated oxidative stress pathways while simultaneously preserving skin barrier homeostasis. These dual therapeutic actions underscore its potential as a cosmeceutical platform for the management of skin diseases.

## Graphical abstract

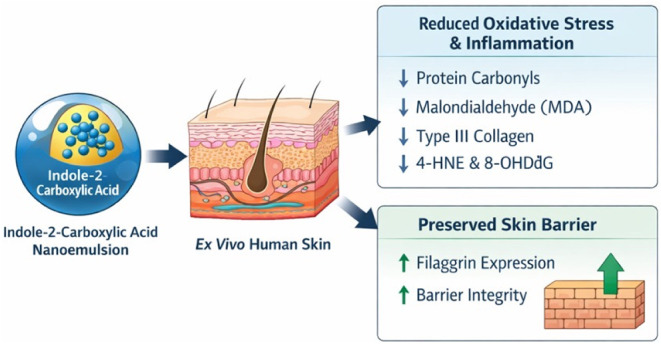

## Introduction

The growing convergence between dermatological research and the cosmeceutical industry has intensified the search for novel, biologically active chemical scaffolds capable of meeting the dual demands of long-term biocompatibility and sustained efficacy in skin-targeted applications.^1^ This effort in driven by the increasing recognition that cutaneous dysfunction and degeneration arise from multifactorial processes integrating environmental exposure, metabolic imbalance, and immune dysregulation, which collectively challenge the performance of conventional single target agents.^2^ Consequently, natural products have regained prominence as a source of structurally diverse and functionally versatile molecules with the potential to address the complex biological landscape of the skin.^3^

Among these bioactive molecules, indole alkaloids have emerged as prominent scaffolds due to their remarkable pharmacological versatility. These compounds exhibit a broad therapeutic spectrum, including treating cancer, cardiovascular diseases, and neurological disorders.^4^ Primarily sourced from terrestrial plants and marine organisms, these alkaloids are increasingly subjected to semisynthetic optimization to enhance potency and selectivity against complex pathologies.^5^ Notably, 2-indoleacetic acid (Figure 1) was first isolated from *Strychnos cathayensis* Merr. (*S. henryi* Merr. & Yamamoto ex Yamamoto), a climbing shrub native to southern China. The roots and seeds of this species are traditionally employed in folk medicine for their antipyretic and hemostatic properties.^6,7^ The 2-indoleacetic acid exhibits significant antioxidant activity by scavenging free radicals and reactive oxygen species (ROS), inhibiting oxidative stress in cells.^8^

**Figure 1:**
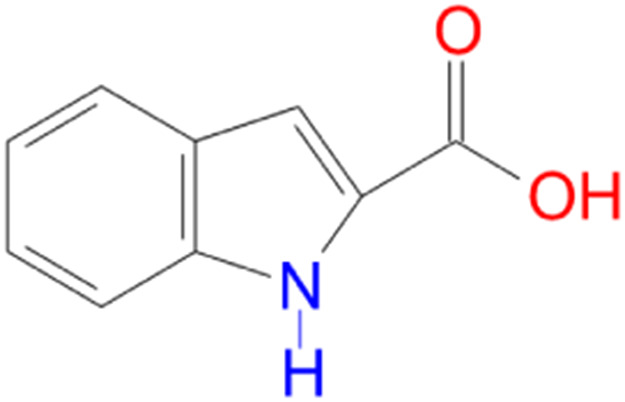
Indole-2-carboxylic acid.

Oxidative stress is defined by a critical imbalance between the production of reactive oxygen species (ROS) and the body’s endogenous antioxidant capacity, acting as a central mechanism in the pathogenesis of various inflammatory skin diseases.^9^ Among the primary exogenous stressors that exacerbate this condition, methylglyoxal (MG) stands out, a highly reactive compound found in cigarette smoke, dietary sources, and rainwater.^10^ The interaction of MG with cellular biomolecules leads to the formation of advanced glycation end-products (AGEs), which trigger an inflammatory cascade characterized by an increase in oxidative damage markers such as 8-OHdG and 4-HNE.^11^ This process establishes a positive feedback loop mediated by the activation of the transcription factor NF-κB, resulting in the degradation of structural markers vital for cutaneous homeostasis, including filaggrin, CD163, and collagen fibers. Based on this, the present investigation proposes the development of an indole-2-carboxylic acid nanoemulsion as an innovative and economically viable therapeutic strategy aimed at modulating oxidative stress and restoring skin barrier integrity.

Building on the well-established anti-inflammatory potential of indole alkaloids and the hemostatic properties attributed to indole-2-carboxylic acid, the present study aimed to develop a nanoemulsion designed to evaluate the anti-inflammatory and antioxidant potential of this compound using an *ex vivo* human skin model. MG, a highly reactive electrophilic α, β-dicarbonyl aldehyde compound, was employed as an inflammation inducer, given that its accumulation disrupts cellular homeostasis and promotes chronic inflammation associated with oxidative stress.^12,13^ Altogether, this research sought to explore the therapeutic relevance of indole-2-carboxylic acid in the treatment of inflammatory skin conditions, recognizing that inflammatory and oxidative processes are dynamically interconnected through a positive feedback cycle.

## Materials and Methods

### Compound

Indole-2-carboxylic acid was purchased from Sigma-Aldrich and used both in its free form (pure compound) and incorporated into nanoemulsion formulations.

### Preparation of Nanoemulsions

The nanoemulsions (NEs) were prepared at room temperature (25 °C) using the spontaneous emulsification method.^14^ Two formulations were developed: (i) a nanoemulsion containing Indole-2-carboxylic acid (N) and (ii) a nanoemulsion without the compound, used as a vehicle control (NS).

Each phase was submitted to the magnetic stirrer for 40 min. The organic phase was then added to the aqueous phase and stirred for 30 min. The resulting solution underwent six cycles in a benchtop disperser. The formulations obtained were designated as N andNS, respectively (Table 1). The components used to produce the nano

**Table 1:** Composition of Nanoemulsion N (Nanoemulsion),NS (Nanoemulsion without the Compound)

	Pharmaceutical Mineral oil^a^	Span 60	Acetone	Compound	Tween 80	Milli-q H_2_O
N	50 mL	36.8 mg	27 mL	40 mg	76.8 mg	53 mL
NS	50 mL	36.8 mg	27 mL	-	76.8 mg	53 mL

a Mineral oil—M3516 Sigma-Aldrich.

### Characterization of Nanoemulsions

#### Particle Size, Span, Polydispersity Index, Zeta Potential, and Encapsulation Efficiency

The particle size, span, polydispersity index, and zeta potential for the NEs were determined using the Zetasizer Nano.^15^ Span, a measure of polydispersity, was calculated from particle diameter measurements corresponding to 10, 50, and 90% (d0.1, d0.5, d0.9, respectively). Particle size and polydispersity index (PDI) were measured by dynamic light scattering (DLS), and zeta potential was measured by electrophoretic mobility (Zetasizer). The samples were diluted in ultrapure water for size measurements and in 10 mmol/L NaCl aqueous solution for zeta potential measurements.

#### *In Vitro* Release Kinetics Study

The release kinetics were determined using the method proposed by Rebekah and coworkers,^16^ with modifications. A cellulose acetate dialysis membrane was placed in a dialysis apparatus, and 1 mL of the N nanoemulsion was added, then sealed. The membrane was immersed in 50 mL of PBS (pH 7.4) as the receptor solution at 37 °C under constant agitation. The procedure was repeated 17 times, and in each repetition, the receptor solution was collected at different times (0, 5, 10, 15, 20, 25, 30, 45, 60, 75, 90, 120, 150, 180, 240, 300, and 360 min). These collected receptor solutions were analyzed using UV–vis Spectrometry.

1 $$\log\;\log\left(q_{e}‐q_{t}\right)=\log\;\log q_{e}‐\frac{k_{1}t}{2.303}$$

2 $$\frac{t}{q_{t}}=\frac{t}{q_{e}}+\frac{1}{K_{2}q_{e}^{2}}$$

q_t_ and q_e_ represent the amount of active ingredient adsorbed (mg g^–1^) at each time (min) and at equilibrium, respectively. K_1_ is the rate constant for the pseudo-first-order reaction (min^–1^), while K_2_ represents the rate constant for the pseudo-second-order reaction (min^–1^).

#### Permeation Studies Using Human Skin

The permeation study of the nanoemulsion through the biological membrane was performed according to the methodology described by Silva and coworkers,^17^ with modifications. The experiment was performed using Franz diffusion cells with a diffusion area of 1.15 cm^2^. Human skin fragments, measuring 12 mm in diameter, were obtained from abdominoplasty procedures using a dermatological punch. Before use, the skin samples were thoroughly cleaned and sterilized and served as the membrane in the diffusion system. In the receiving compartment, 2 mL of PBS (pH 7.4) was added, and in the donor compartment, 1.0 mL of the nanoemulsion suspension was added. The system was maintained at 37 °C and agitated for 26 h. The membrane was placed on top of the receiving cell. At the end of the incubation period, the membrane was collected and digested. Skin samples were subjected to an ultrasonic treatment for 1 h in 4 mL of acetonitrile, followed by vertexing for 3 min and centrifugation for 15 min. The receptor solution was analyzed directly. Compound concentrations in both the receptor solution and the membrane were quantified using UV–vis at 280 nm. All experiments were conducted in triplicate.

### *In Vitro* Analyses

#### Cell Viability

Macrophages were seeded at a density of 1 × 10^5^ cells per well in 200 μL of RPMI medium supplemented with 10% fetal bovine serum (FBS) in 96-well plates and incubated for 24 h at 37 °C with 5% CO_2_. After incubation, 50 μL of supernatant was removed and replaced with fresh medium containing the treatments. Serial dilutions of the pure compound (Indole-2-carboxylic acid) were prepared to obtain final concentrations of 100, 50, and 25 μg/mL. The blank nanoemulsion (NS) was serially diluted starting at 100 μg/mL to obtain final concentrations of 100, 50, 25, 12.5, 6.25, and 3.125 μg/mL. The compound-loaded nanoemulsion (N) was evaluated at its original formulation concentration (0.5 mg/mL; 40 mg in 80 mL), without further dilution, to assess potential cytotoxic effects under maximal exposure conditions. Plates were incubated for an additional 48 h under the same conditions. Two controls were included: (Control 1) cells cultured in RPMI medium and (Control 2) cells treated with RPMI containing 1% DMSO to confirm solvent nontoxicity. After treatment, 50 μL of supernatant was removed and replaced with 50 μL of MTT solution (0.05 mg/mL). Plates were incubated for 3 h, after which the formazan crystals were solubilized with DMSO, and absorbance was measured at 570 nm.

#### Cell Viability Analyses After LPS (Lipopolysaccharide) Exposure

Macrophages were seeded at 1 × 10^5^ cells per well in 200 μL of DMEM supplemented with 10% FBS in 96-well plates and incubated for 24 h at 37 °C and 5% CO_2_. A solution of LPS was prepared by mixing 400 μL of LPS (218 mM) with 19.6 mL of DMEM, from which the test compound was solubilized at 100 μg/mL. The culture medium was removed, and treatments were added. The experimental controls were: Control 1 – Cells + Medium (blank); Control 2 – Cells + Medium + LPS (positive); Control 3 – Dexamethasone (100 μg/mL). Plates were incubated for an additional 24 h. After incubation, 50 μL of supernatant was collected from each well for NO quantification. A revealing solution containing 1% sulfanilamide and 0.1% N-(1-naphthyl)­ethylenediamine dihydrochloride (1:1) was added (100 μL/well) and incubated for 15 min in the dark. A standard curve starting at 0.15 mM was prepared for quantification. Absorbance was measured at 540 nm.

#### *Ex Vivo* Analysis with Human Skin Biopsies

Human skin samples were obtained with informed consent from patients undergoing plastic surgery in Viçosa, MG, Brazil, in accordance with the ethical guidelines of the Human Research Ethics Committee of the Federal University of Viçosa (CAAE: 82329224.7.0000.5153). Human skin fragments were obtained from abdominoplasty procedures. The subcutaneous tissue was removed to preserve the dermis and epidermis, and the skin explants were prepared using a 12 mm dermatological punch. Each fragment was subsequently washed in sterile PBS buffer and transferred to a six-well plate and cultured in DMEM High Glucose medium (GIBCO, USA) supplemented with 1% Fetal Bovine Serum (GIBCO, USA), 1% liquid penicillin, and 2.5 μg/mL amphotericin B. The samples were incubated for 24 h at 37 °C in a 5% CO2 atm to allow tissue recovery.^18^ The explants received daily topical treatments for 7 days with DMEM High Glucose medium containing 650 μM Methylglyoxal (MGO). The experimental groups were defined as follows: **C– (negative control)**: explants treated with DMEM medium only, without MG exposure, **C+ (positive control)**: explants exposed to 650 μM methylglyoxal, **NS**: explants exposed to 650 μM methylglyoxal and treated with 20 μL of the nanoemulsion without the active ingredient, **N**: explants exposed to 650 μM methylglyoxal and treated with 20 μL of the nanoemulsion containing the active ingredient. The culture medium was replaced daily, and all experiments were conducted in quadruplicate. At the end of the treatment period, the explants were collected and divided into two portions: one was fixed in formalin, embedded in paraffin, and processed for histological and immunohistochemical analysis; the other was frozen in liquid nitrogen for subsequent evaluation of oxidative stress products and enzymatic activities.

#### Antioxidant Enzyme Activity Analysis

After 7 days of NS treatment, the skin fragments designated for oxidative stress analysis were frozen in liquid nitrogen (−196 °C) and stored at −80 °C until further analysis. The samples were weighed and manually ground in liquid nitrogen with a pestle, then homogenized in phosphate-EDTA buffer (0.2 mol/L phosphate buffer and 1 mmol/L ethylenediaminetetraacetic acid, pH 7.4). Following homogenization, the samples were centrifuged at 15,000 g for 10 min at 4 °C. The resulting supernatants were used to determine the activities of the antioxidant enzymes superoxide dismutase (SOD), catalase (CAT), and glutathione S-transferase (GST).

The method proposed by Dieterich and co-workers^19^ was used for SOD quantification activity. The assay is based on the ability of SOD to eliminate the superoxide anion radical (O_2_^–^), thus reducing the overall rate of autoxidation of pyrogallol. An ELISA plate was used, in which 30 μL of the sample was placed, 99 μL of potassium phosphate buffer (0.2 M, pH 8), and 144 μL of potassium phosphate buffer (0.2 M, pH 8) was used as a blank, and 129 μL of potassium phosphate buffer (0.2 M, pH 8) was used as a control. Six μL of MTT (3-[4,5-dimethylthiazole-2H]-2,5-diphenyltetrazolium bromide) was pipetted into all wells, then 15 μL of Pyrogallol was pipetted into all wells, except the blank. The plate was incubated for 15 min at 40 °C in the oven. Then 150 μL of dimethyl sulfoxide (DMSO) was pipetted into all wells to stop the reaction. The reading was performed at 540 nm.

CAT activity was evaluated using the Aebi method,^20^ which measures the ability to decompose hydrogen peroxide into water and oxygen. In an ELISA plate, 5 μL of the supernatant (sample) and 100 μL of hydrogen peroxide (20 mM) were placed. The control sample was composed of 5 μL of the supernatant (sample) and 100 μL of potassium phosphate buffer (50 mM, pH 7.4). The standard curve was prepared using 5 μL of potassium phosphate buffer (50 mM, pH 7.4) and 100 μL of hydrogen peroxide solutions with concentrations ranging from 0 to 20 mM. The blank was prepared with 100 μL of potassium phosphate buffer (50 mM, pH 7.4). After 3 min, 150 μL of ammonium molybdate (32.4 mmol/L) was added to all wells. The absorbance was read at 374 nm.

For GST, the method of Habig and co-workers^21^ with adaptations was used. Enzyme activity was evaluated using 1-chloro-2,4-dinitrobenzene (CDNB) as the substrate. The reaction mixture contained 970 μL of potassium phosphate buffer (pH 7), 10 μL of CDNB, 10 μL of homogenate, and 10 μL of GSH. The blank consisted of 950 μL of potassium phosphate buffer (pH 7), 50 μL of CDNB, and 50 μL of GSH. Absorbance readings were performed at 340 nm at 0, 30, 60, and 90 s.

#### Oxidative Stress Markers

After centrifugation of the skin fragments treated with nanoemulsions, the resulting pellets were used to analyze carbonylated protein (CP) and malondialdehyde (MDA). CP levels were determined using the method proposed by Levine.^22^ Results were normalized to total protein concentration in the supernatant, according to the method described by Lowry and coworkers.^23^

A volume of 500 μL of 10% trichloroacetic acid (TCA) was added to an Eppendorf tube containing the pellet, followed by homogenization. An additional 500 μL of 10% TCA was then added, and the mixture was homogenized once more. The contents were divided into two tubes (A and B), which were centrifuged at 3000 rpm for 10 min at 25 °C. After discarding the supernatants, 500 μL of 10 mM 2,4-dinitrophenylhydrazine (DNPH) was added to tube A, while tube B received 500 μL of HCl. The tubes were homogenized and incubated in the dark for 30 min, with additional homogenization every 15 min. Subsequently, they were centrifuged at 3000 rpm for 10 min at 25 °C, and the supernatants were discarded. A volume of 500 μL of 10% TCA was added to both tubes, and the mixture was homogenized. The tubes were centrifuged again at 3000 rpm for 10 min at 25 °C, and the supernatants were discarded. The precipitates were washed with 1 mL of ethanol:ethyl acetate mixture, homogenized until the pellet was fully suspended, followed by centrifugation at 3000 rpm for 10 min at 25 °C and discarding the supernatant. This process was carried out 3 times. The final precipitate was dissolved in 1 mL of 6% sodium dodecyl sulfate (SDS) solution, homogenized, and heated in a double boiler for 30 min at 32 °C. Afterward, the samples were centrifuged at 6000 rpm for 10 min at 25 °C. Finally, 200 μL of the supernatant was transferred to an ELISA plate, and the absorbance was measured at 370 nm.

The MDA levels were determined based on the method described by Wallin and coworkers,^24^ which involves the reaction of thiobarbituric acid reactive substances (TBARS) with MDA to form a colored complex. For each sample, 200 μL was added to microtubes, followed by 400 μL of the TBARS solution and vortex homogenization. The mixture was then incubated in a double boiler at 90 °C for 40 min. Thereafter, tubes were cooled in an ice bath for 5 min, and 600 μL of n-butanol was added. The samples were vortexed for 1–2 min until phase inversion occurred, then centrifuged at 3500 rpm for 10 min at 15 °C. Finally, 200 μL of the upper organic phase was transferred to an ELISA plate, and absorbance was measured at 540 nm.

#### Histological Evaluation

Paraffin-embedded sections, 5 μm thick, were obtained using a Leica Multicut 2045 microtome (Reichert–Jung Products, Germany) and deposited on histology slides. Histopathological analyses were carried out to evaluate the effectiveness of nanoemulsion (N) compared with the nanoemulsion without the active ingredient (NS). The following stains were used: Hematoxylin–Eosin (HE) to analyze the thickness of the epidermis and quantify blood vessels,^25^ Sirius Red staining to differentiate and quantify type I and type III collagen fibers,^26^ and Toluidine Blue staining to identify mast cells.^27^ A photomicroscope (EVOS M5000 Imaging System, Thermo Fisher Scientific) equipped with a digital camera (3-QColor Olympus) was used. The images were analyzed using Image-Pro Plus 4.5 (Media Cybernetics, Silver Spring, USA) to manually count tissue features on a 300-point grid. Epidermal thickness was measured in micrometers (μm). Histological sections were photographed using a 40x objective lens. The histopathological analyses were performed in triplicate.

#### Immunohistochemical Evaluation

Immunohistochemical assays were performed to investigate the potential protective effect of the nanoemulsion (N) against oxidative damage and inflammation, using markers of skin integrity and oxidative stress, as described by Morais-Santos and coworkers.^28^ The 5-μm paraffin-embedded sections were deparaffinized, rehydrated, blocking of endogenous peroxidase. Antigen retrieval was performed by heating the sections in 0.1 M sodium citrate buffer (pH 6.0) for 20 min in a microwave. To prevent nonspecific binding, the sections were incubated with 5% BSA for 1 h in a humid chamber. After removing the BSA, primary antibodies were applied, including antifilaggrin (1:250), anti-8OHdG (1:50), anti-CD163 (1:50), and anti-4HNE (1:500). The sections were incubated overnight in a humidity chamber at 4 °C. For negative controls, only PBS was used. Following the removal and washing of the primary antibodies, the sections were incubated with secondary antibodies—antimouse (1:600), antimouse (1:600), antirabbit (1:200), and antigoat (1:50), respectively, for 1 h in the dark. Subsequently, the slides were immersed in a developer solution containing 0.05% DAB (3,3′-diaminobenzidine tetrahydrochloride) and 0.01% hydrogen peroxide in Tris-HCl buffer (0.05 M, pH 7.4). The sections were then counterstained with Harris Hematoxylin, followed by dehydration and slide mounting.

### Statistical Analysis

Statistical analyses were conducted using GraphPad Prism version 8 (GraphPad Software Inc., Boston, MA, USA). Data are presented as mean ± standard deviation (SD). Group comparisons were performed using one-way analysis of variance (ANOVA) followed by the Newman–Keuls post hoc test. A p-value of <0.05 was considered statistically significant.

## Results

### Characterization of Nanoemulsions

#### Particle Size, Span, Polydispersity Index, Zeta Potential, and Encapsulation Efficiency

Characterization of nanoemulsion enables assessment of its safety, efficacy, and stability based on its physicochemical properties. Accordingly, parameters such as zeta potential, average particle size, and polydispersity index (PDI) are essential for the development of pharmaceutical formulations (Table 2, Figure 2). The average particle sizes, determined by dynamic light scattering (DLS), were 129.03 nm for formulation N and 131.58 nm for NS. These values are consistent with nanostructured systems, as they are below 200 nm, indicating potential for applications that require good permeation. Formulations with zeta potentials between ±10 mV and ±30 mV are considered reasonably stable when nonionic surfactants such as Tween 80 are used in these formulations. The NS nanoemulsion presented a more negative zeta potential, suggesting greater stability compared to nanoemulsion N.

**Table 2:** Physicochemical Characteristics of the N and NS Nanoemulsion

Formulation	Size (nm)	PDI	Zeta potencial (mV)	Encapsulation efficiency (%)
N	129.03	8.90	–15.45	
NS	131.58	11.90	–16.69	96

**Figure 2:**
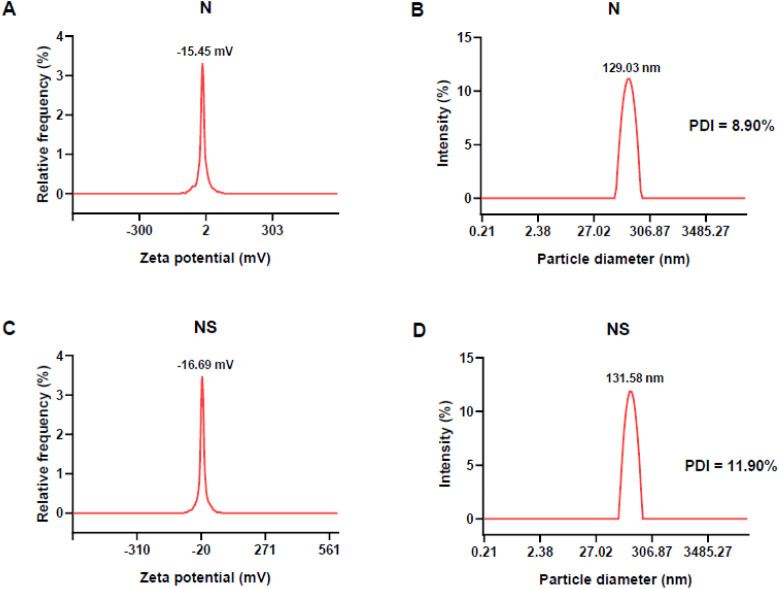
Physicochemical characteristics of the N and NS nanoemulsion. (**A**) Zeta potential plot for the N nanoemulsion; (**B**) Particle size plot for the N nanoemulsion; (**C**) Zeta potential plot for the NS nanoemulsion; (**D**) Particle size plot for the NS nanoemulsion.

The polydispersity index (PDI), which reflects the uniformity of particle size distribution, was 8.9% for N and 11.9% for NS. A PDI below 10% is considered ideal and indicates a homogeneous distribution, as observed for formulation N; however, a PDI of 11.9% may also be interpreted as indicative of good uniformity. Overall, the NS nanoemulsion exhibited good stability, uniformity, and permeability.

#### *In Vitro* Release Kinetics Study

A kinetic release study of 2-indoleacetic acid was conducted to characterize the nanoemulsion as a biomaterial and provide insights into its functional behavior. Based on the kinetic parameters, especially the R2 value, the release of the active ingredient from the nanoemulsion follows a pseudo-second-order model, with a higher R2 (0.970) than the pseudo-first-order model (0.929).

The results, presented in Figure 3A, show a rapid release of the drug during the first hour (0.017 mg L^–1^), followed by a slower and sustained release (0.020 mg L^–1^) during subsequent hours.

**Figure 3:**
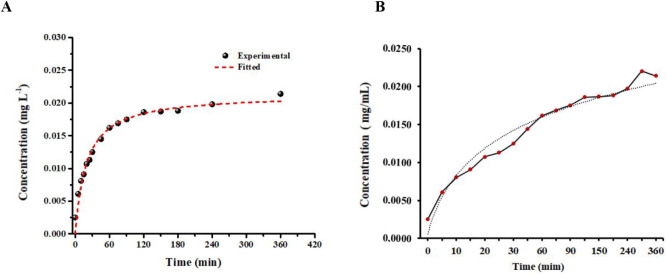
**A**) Represents the normalized release kinetics of nanoemulsion containing the active ingredient (N). Fitted: kinetic release profile. Experimental: kinetic release profile of (N); **B**) Represents the normalized release kinetics of compound 2-indole-acetic acid in a biomimetic membrane over 360 min. The compound demonstrated rapid penetration through the biomimetic membrane, with progressively increasing levels in the receptor solution over time, highlighting its high permeation capacity. In the initial minutes, the permeation curve shows a lower release of the compound, which reaches its maximum concentration of around 300 min. Different letters in the columns indicate statistically significant differences (*P* < 0.05) between the groups, according to the Newman-Keuls test.

#### Permeation Studies Using Human Skin

The results of the nanoemulsion (N) permeation through biological barriers, using explanted human skin as a membrane model, are presented in Figure 4. Of the total compound applied (0.48 mg L^–1^), 37,14% remained retained in the stratum corneum, while 43.98% was recovered in the receptor solution after 26 h. These findings demonstrate that the compound dispersed in the nanoemulsion was able to effectively traverse the skin barrier and reach the receptor compartment, indicating the formulation’s potential for transdermal delivery.

**Figure 4:**
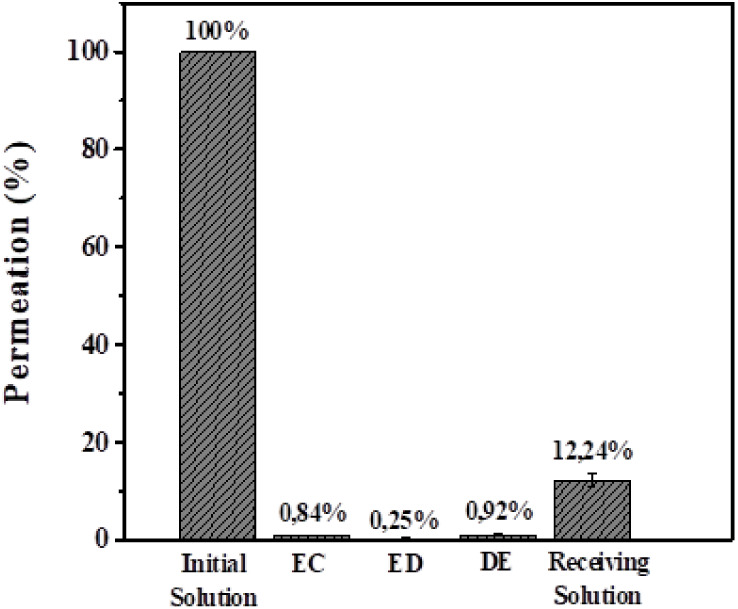
Permeation in explanted human skin, simulating a real biological barrier EC (Stratum Corneum); ED (Epidermis); DE (Dermis).

#### Cell Viability of 2-Indoleacetic Acid

The compound showed no cytotoxic effect on RAW 264.7 macrophages at 100, 50, and 25 mg/mL (Figure 5A), indicating the absence of toxicity to these cells. In the nitric oxide assay, the group exposed to LPS (C2) showed an increase in NO production, indicating oxidative stress and inflammation in the cells. Additionally, there was a reduction in nitric oxide after exposure to 100 μg/mL. The decrease after 100 μg/mL exposure was more significant compared to the dexamethasone group, as shown in Figure 5B.

**Figure 5:**
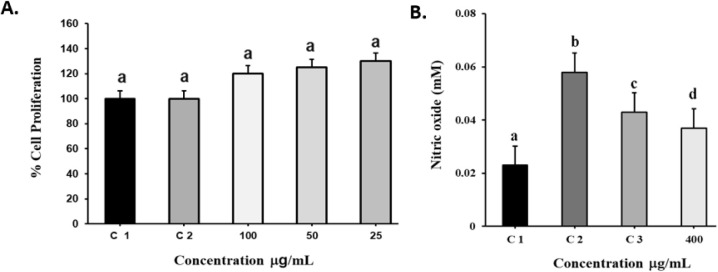
A) Cell viability in RAW 264.7 macrophages. Data represent triplicate means from three biological replicates and are expressed as percent viability. Controls: C1 (Cells + Medium); C2 (Cells + Medium +0.01% DMSO). Different letters denote significant differences. B) Nitric oxide (NO) reduction in RAW 264.7 macrophages treated with compound indole-2-carboxylic acid (100 μg/mL). Data represent triplicate means and are expressed as NO reduction (mM). Controls: C1 (Cells + Medium); C2 (Cells + Medium + LPS); C3 (Cells + Medium + LPS + Dexamethasone); I2AC (indole-2-carboxylic acid). Different letters indicate significant differences^.a,b,c,d^ Different letters in the columns indicate statistically significant differences (*P* < 0.05) between the groups, according to the Newman-Keuls test.

#### Cell Viability of Nanoemulsions NS and N

Neither the blank nanoemulsion (NS) nor the compound-loaded nanoemulsion (N) showed cytotoxicity toward RAW264.7 macrophages under the tested conditions (Figure 6). In Figure 6A, the blank nanoemulsion (NS) maintained cell viability with a modest increase in MTT signal observed at 50, 25, and 12.5 μg/mL compared with the controls. In Figure 6B, the compound-loaded nanoemulsion (N), evaluated at its original formulation concentration (0.5 mg/mL), also did not reduce cell viability.

**Figure 6:**
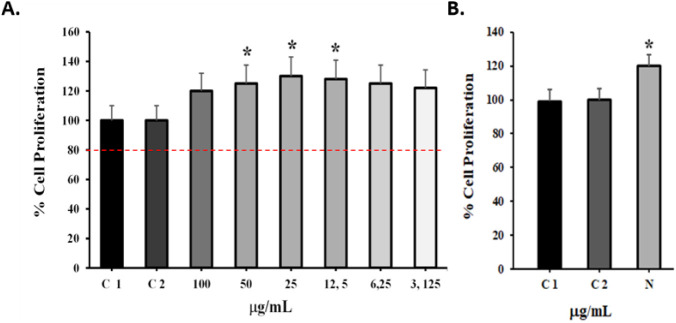
Evaluation of cell viability under the macrophage cell line RAW 264.7. (A) Blank nanoemulsion (NS) tested at concentrations ranging from 100 to 3.125 μg/mL. (B) Compound-loaded nanoemulsion (N) evaluated at its original formulation concentration (0.5 mg/mL). Results were calculated in triplicate, and the experiment was performed with three biological replicates. Data were calculated as a percentage of cell viability. Control 1 (C1) (Cells + Medium); Control (C2) (Cells + Medium + DMSO at 0.01%). The asterisks (*) indicate statistically significant differences (*P* < 0.05) between the groups, according to the Newman-Keuls test.

### *Ex Vivo* Studies in Human Skin Explant Culture

#### Antioxidant Enzyme Activity Analysis

In the analysis of Superoxide Dismutase antioxidant enzymes (Figure 7A), the groups treated with the nanoemulsion without the active ingredient (NS) and nanoemulsion containing the active ingredient (N) resulted in a reduction of this enzyme activity compared to C+ (mg/ptn). N nanoemulsion was more effective in reducing SOD activity than NS nanoemulsion. Nanoemulsion containing the active ingredient (N) was more effective in reducing CAT than nanoemulsion without the active ingredient (NS), and both were effective when compared to the C+ (U/mg ptn). Treatments with the nanoemulsion without the active ingredient (NS) and nanoemulsion containing the active ingredient (N) resulted in a reduction in glutathione-S-transferase activity (units per mg of protein) compared to C+; however, no significant difference was observed between the two nanoemulsions regarding glutathione activity (Figure 7C).

**Figure 7:**
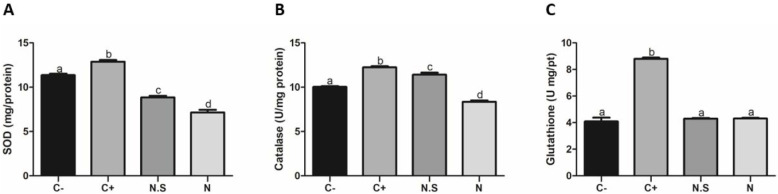
Antioxidant enzyme activity: Superoxide Dismutase (SOD) (**A**), Catalase (CAT) (**B**), and Glutathione S-Transferase (GST) (**C**). C–: explants not exposed to methylglyoxal, C+: explants exposed to 650 μM methylglyoxal, NS: explants exposed to 650 μM methylglyoxal and treated with nanoemulsion without the active ingredient, N: explants exposed to 650 μM methylglyoxal and treated with nanoemulsion containing the active ingredient. ^a,b,c,d^Different letters in the columns indicate statistically significant differences (*P* < 0.05) between groups, according to the Newman-Keuls test.

#### Oxidative Stress Markers

In Figure 8, we can observe that the treatments with nanoemulsions (NS and N) significantly reduce the MDA concentration and carbonylated proteins, compared to C+ (*p* < 0.05). It is important to highlight that C+, NS and N were stressed by methylglyoxal exposure.

**Figure 8:**
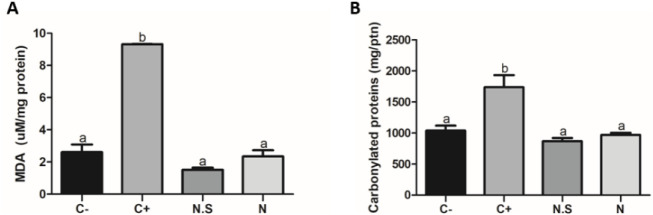
Malondialdehyde (MDA) (**A**) and carbonylated proteins (**B**). C–: explants not exposed to methylglyoxal, C+: explants exposed to 650 μM methylglyoxal, NS: explants exposed to 650 μM methylglyoxal and treated with nanoemulsion without the active ingredient, N: explants exposed to 650 μM methylglyoxal and treated with nanoemulsion containing the active ingredient. ^a,b^ Different letters in the columns indicate statistically significant differences (*P* < 0.05) between groups, according to the Newman–Keuls test.

#### Histopathological Evaluation

Histopathological evaluation revealed no significant differences in epidermal thickness (Figure 9A–B) among the groups. However, the treatments with nanoemulsions without the active ingredient (NS) and containing the active ingredient (N) significantly reduced the blood vessels when compared to the C+ (*p* < 0.05) (Figure 9C).

**Figure 9:**
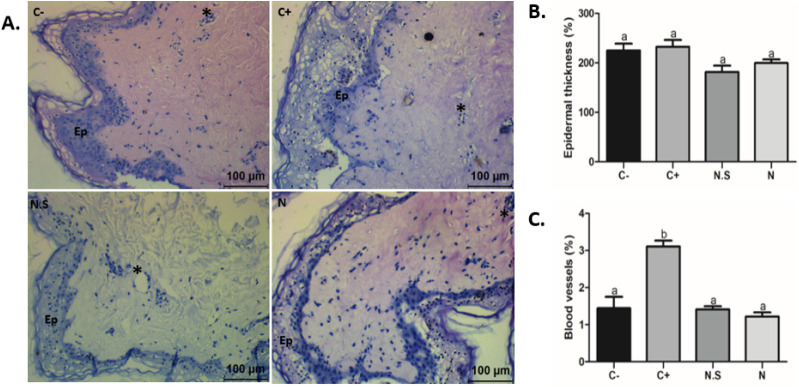
A. Photomicrograph of epithelial tissue stained with Hematoxylin and Eosin for the analysis of epidermal thickness, and blood vessels percentage. C–: explants not exposed to methylglyoxal, C+: explants exposed to 650 μM methylglyoxal, NS: explants exposed to 650 μM methylglyoxal and treated with nanoemulsion without the active ingredient, N: explants exposed to 650 μM methylglyoxal and treated with nanoemulsion containing the active ingredient. Ep= epidermis, Asterisk= blood vessels. Epidermal thickness (**B**), and blood vessels percentage (**C**). C–: explants not exposed to methylglyoxal, C+: explants exposed to 650 μM methylglyoxal, NS: explants exposed to 650 μM methylglyoxal and treated with nanoemulsion without the active ingredient, N: explants exposed to 650 μM methylglyoxal and treated with nanoemulsion containing the active ingredient. ^a,b^ Different letters in the columns indicate statistically significant differences (*P* < 0.05) between the groups, according to the Newman-Keuls test.

When analyzing the percentage of mast cells, it revealed no significant differences in mast cell percentage among the groups (Figure 10).

**Figure 10:**
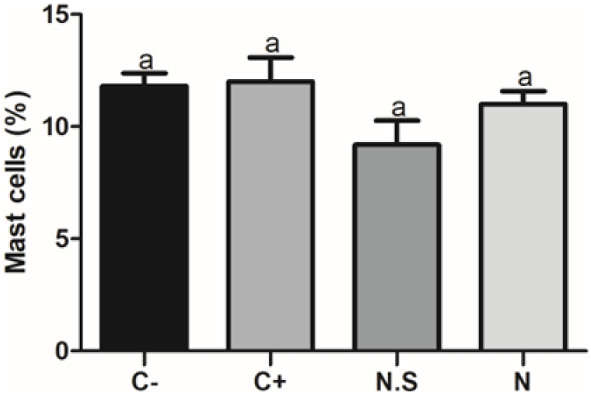
Histological evaluation. Mast cells percentage. C–: explants not exposed to methylglyoxal, C+: explants exposed to 650 μM methylglyoxal, NS: explants exposed to 650 μM methylglyoxal and treated with nanoemulsion without the active ingredient, N: explants exposed to 650 μM methylglyoxal and treated with nanoemulsion containing the active ingredient. Groups with the same letter do not show a statistically significant difference (*P* > 0.05), according to the Newman-Keuls test.

In the histological analysis of type I collagen, we observed that the groups treated with methylglyoxal (C+, NS, N) showed a reduction in the percentage of collagen fibers when compared to the group not exposed to methylglyoxal (C−) (Figure 11A, B). In the analysis of type III collagen, we observed that there was a significant reduction in nanoemulsions (NS and N) compared to the C+ (Figure 11A, C). The photomicrographs of epithelial tissue indicating the presence of type I collagen fibers and type III collagen fibers are shown in Figure 11.

**Figure 11:**
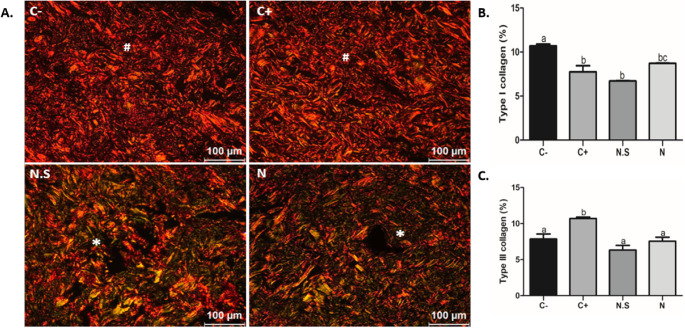
Photomicrograph of epithelial tissue stained with Sirius Red to analyze type I and type III collagen fibers (**A**).C–: explants not exposed to methylglyoxal, C+: explants exposed to 650 μM methylglyoxal, NS: explants exposed to 650 μM methylglyoxal and treated with nanoemulsion without the active ingredient, N: explants exposed to 650 μM methylglyoxal and treated with nanoemulsion containing the active ingredient. The Yellow-red color indicates the presence of type I collagen, and the green color indicates the presence of type III collagen. Hashtag= type I collagen fibers and Asterisk = type III collagen fibers. Histological evaluation. Type I collagen fibers percentage (**B**) and type III collagen fibers percentage (**C**). C–: explants not exposed to methylglyoxal, C+: explants exposed to 650 μM methylglyoxal, NS: explants exposed to 650 μM methylglyoxal treated with nanoemulsion without the active ingredient, N: explants exposed to 650 μM methylglyoxal treated with nanoemulsion containing the active ingredient. ^a,b,c^ Different letters in the columns indicate statistically significant differences (*P* < 0.05) between the groups, according to the Newman-Keuls test.

#### Immunohistochemical Evaluation

For the immunohistochemical assay, the treatments with the nanoemulsions (NS and N) significantly increased filaggrin levels compared to the C+ group (Figure 12A). The nanoemulsions significantly increased CD163 levels compared to the C+ group, with the nanoemulsion containing the active ingredient showing the most pronounced effects (Figure 12B). However, no difference in 8-OHdG levels was observed among the groups (Figure 12C). The nanoemulsions (NS and N) decrease 4HNE levels compared to the C+ group, with the nanoemulsion without an active ingredient showing the most pronounced effects (Figure 12D). Immunohistochemical evaluation indicating the presence of filaggrin, 8-OHDG, CD-163, and 4HNE markers is shown in Figure 13.

**Figure 12:**
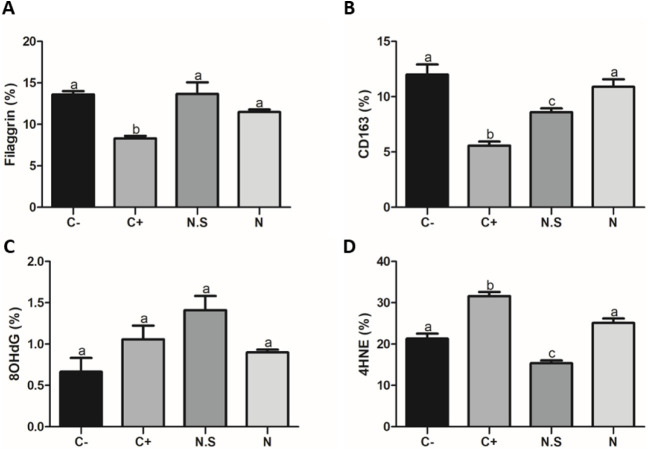
Immunohistochemical evaluation of filaggrin (A), CD163 (B), 8OHdG (C), and 4HNE (D). C–: explants not exposed to methylglyoxal, C+: explants exposed to 650 μM methylglyoxal, NS: explants exposed to 650 μM methylglyoxal treated with nanoemulsion without the active ingredient, N: explants exposed to 650 μM methylglyoxal treated with nanoemulsion containing the active ingredient. ^a,b,c^ Different letters in the columns indicate statistically significant differences (*P* < 0.05) between the groups, according to the Newman-Keuls test.

**Figure 13:**
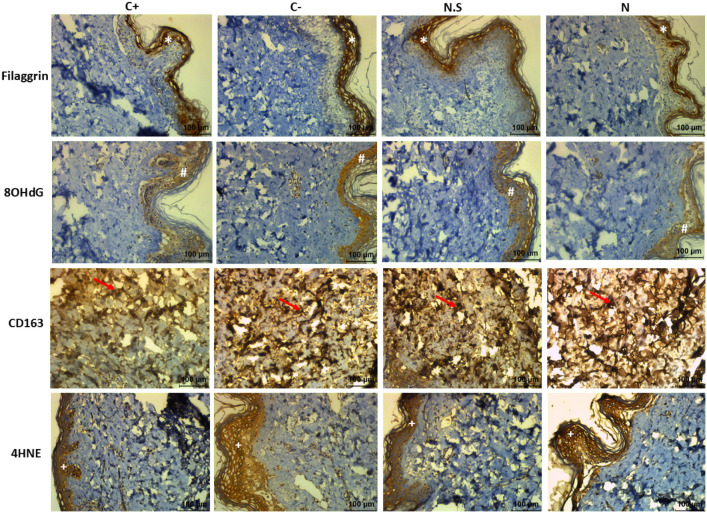
Immunohistochemical evaluation for filaggrin, 8OHdG, CD163, and 4HNE. C–: explants not exposed to methylglyoxal, C+: explants exposed to 650 μM methylglyoxal, NS: explants exposed to 650 μM methylglyoxal treated with nanoemulsion without the active ingredient, N: explants exposed to 650 μM methylglyoxal treated with nanoemulsion containing the active ingredient. Asterisk = filaggrin, Hashtag = epithelial cells, Arrows = macrophages, Plus sign= 4HNE marker.

## Discussion

Skin conditions are among the most prevalent dermatological disorders, characterized by chronic, proliferative processes influenced by both genetic predisposition and environmental triggers, such as UV exposure and pollutants.^29^ In this context, topical formulations with antioxidant potential are of significant interest in cosmetic science, particularly those leveraging nanotechnology for improved skin penetration and therapeutic performance. Given this landscape, developing effective therapies to control inflammation and mitigate oxidative damage is crucial. Nanoemulsions have emerged as a promising strategy alongside the search for novel bioactive compounds. In this study, In the present study, we utilized the synthetic alkaloid indole-2-carboxylic acid, inspired by literature reports describing the anti-inflammatory properties of indole alkaloids.^30^ Previous studies have demonstrated that indole-2-carboxamide derivatives represent promising anti-inflammatory compounds, capable of reducing several inflammatory mediators. In addition, N-substituted indole-2-carboxylic acid derivatives have also been investigated for their antioxidant potential^31,32^ and formulated a nanoparticle-based drug delivery system. With that in mind, this study aims to explore the development and *ex vivo* biological evaluation of a nanoemulsion containing indole-2-carboxylic acid, an indole alkaloid, to avoid oxidative stress and skin damage.

The formulation was prepared with high efficiency (96%). The ingredients used in the composition of the nanoemulsion were carefully selected based on their widespread pharmaceutical application and suitability for topical use. The nonionic surfactants Span 60, and Tween 80 were chosen due to their high biocompatibility, extensive use in pharmaceutical formulations, and relatively low irritancy profile for topical applications. Acetone was used only as a cosolvent to assist in compound solubilization and was completely evaporated during the nanoemulsion preparation process. The characterization was obtained by dynamic light scattering (DLS), which revealed a mean hydrodynamic diameter of 131.58 nm and a narrow polydispersity index (span = 0.65), indicating a uniform particle size distribution suitable for dermal application. Zeta potential analysis (−16.69 mV) with acceptable colloidal stability, showing no aggregation during storage.^33^ The *in vitro* release profile of the active compound from the nanoemulsion demonstrated a biphasic pattern, consisting of an initial burst phase followed by a sustained release, consistent with pseudo-second-order kinetics (R^2^ = 0.970). Such behavior is advantageous for topical delivery systems, allowing rapid onset of action followed by prolonged activity at the site of application. Skin permeation studies using *ex vivo* human skin showed that approximately 44% of the applied dose permeated the membrane over 26 h, while 37% remained retained in the stratum corneum. This dual behavior supports both immediate and depot-like actions of the formulation, an important feature for cosmetics and dermocosmetics aimed at maintaining skin homeostasis and preventing flare-ups of inflammatory episodes. These findings are consistent with previous studies that emphasize the enhanced skin penetration of nanostructured carriers.^34^

After observing that the nanoemulsion containing indole-2-carboxylic acid exhibited favorable skin permeation in human samples, the next stage was to assess its cytotoxicity. Results indicated that cell viability actually increased following exposure to concentrations of 50, 25, and 12.5 μg/mL. This suggests that indole-2-carboxylic acid is not only nontoxic but may also enhance cellular metabolism. Notably, this metabolic boost was maintained even when the compound was delivered via nanoemulsion. Similar increases in cell viability have been reported for other bioactive compounds delivered via nanoemulsions, including plant-derived antioxidants and indole derivatives, which enhanced mitochondrial activity without inducing cytotoxicity.^35,36^ The cell viability analyses were also important because they helped us determine the best concentration to use in the next steps of the experiment.

In the next step, in alignment with global pressure to reduce reliance on animal models and replace them in pharmacological research, this study employed an *ex vivo* methodology. Human tissues allow researchers to obtain robust data before moving on to animal testing, and it is easier to justify the use of tissue that would be discarded in surgery (such as biopsies or excess skin from plastic surgery) than to create and sacrifice laboratory animals. Furthermore, this model enables the direct observation of how human tissue responds to oxidative stress and inflammation following exogenous exposure. Unlike murine models, the *ex vivo* approach utilizes human genetics and physiology, effectively overcoming the “species barrier”. Given that many therapeutic compounds fail in clinical trials due to distinct metabolic pathways and receptor affinities in humans compared to animals, this model represents a promising and highly desirable tool for the development of novel therapeutics. Considering the ability of methylglyoxal to induce oxidative stress^13,37^ and disrupt the cellular environment, leading to oxidative imbalance,^38^ we evaluated how stressed tissue treated with nanoemulsions containing or not containing synthetic alkaloid indole-2-carboxylic acid affected antioxidant enzyme activity (SOD, CAT and GST) and oxidative stress markers (carbonylated proteins and MDA). We observed an increase in oxidative stress markers in the groups exposed to methylglyoxal, whereas treatment with the nanoemulsions reduced these markers and modulated antioxidant enzyme activity. We hypothesize that this reduction in oxidative stress decreased the demand for high antioxidant enzyme activation, contributing to redox balance and cellular protection. This was further corroborated by the reduction in malondialdehyde (MDA) and protein carbonyl levels, particularly in the N group, indicating attenuated lipid and protein oxidative damage. Although the blank nanoemulsion (NS) also exhibited relevant antioxidant activity, the formulation containing indole-2-carboxylic acid generally promoted a more consistent protective effect when the results were analyzed collectively. These findings suggest that both the nanoemulsion system and the active compound contribute to the biological activity observed, possibly through synergistic mechanisms and improved tissue interaction provided by the nanocarrier system.^39^

Following the assessment of antioxidant enzyme activity and oxidative stress markers, we conducted histological and immunohistochemical analyses to evaluate structural tissue alterations and cellular responses to treatment. A significant increase in blood vessel density was noted in the C+ group; conversely, the nanoemulsion-treated groups (N and NS) maintained vessel counts similar to the C– group. While angiogenesis is a physiological process, it can become pathological when driven by pro-inflammatory cytokines, as seen in conditions like psoriasis and rosacea.^40^ Thus, we hypothesize that nanoemulsions protect the skin from exacerbated angiogenesis, maintaining vascular levels comparable to normal tissue. These results align with previous studies where nanoemulsions significantly reduced vessel overproliferation,^41^ likely due to their small particle size and superior permeation.^42^ Regarding the inflammatory response, mast cell counts showed no significant differences between groups, suggesting that neither methylglyoxal (MG) nor the nanoemulsions influenced mast cell recruitment or degranulation under the evaluated conditions.

As a highly reactive agent, MG promotes the formation of Advanced Glycation End Products (AGEs), which damage skin proteins and impair their functions.^43^ Our results show that MG (C+ group) reduced the percentage of type I collagen fibers compared to the negative control. Since nanoemulsion N did not significantly reverse this effect, it likely acts through inflammatory pathways rather than antiglycation. Regarding type III collagen, a key component of granulation tissue, we observed increased levels in the C+ group, reflecting a response to MG-induced damage.^44^ The N-treated group, however, showed levels comparable to the C– group. This indicates that nanoemulsion helps regulate the inflammatory pathways and repair response, preventing overdeposition of type III collagen and fibrosis in the tissue.

In the immunohistochemistry assay, we analyzed the filaggrin marker, a key structural protein in the skin’s barrier function, which contributes to the physical resistance of the stratum corneum and is a source of hydration, combining with other hydrophilic components to keep the skin hydrated.^45,46^ We observed a decrease in the percentage of filaggrin in the C+, while no change was observed in the nanoemulsions when compared to the C–. However, it is important to keep intracellular levels of filaggrin safe, when there is too much profilaggrin in the cytosol, this excess causes cytotoxicity and cell death, leading to a dysfunction in the skin barrier.^47^ Our nanoemulsion protected the cells from excess filaggrin, maintaining similar levels when compared to C–, we believe that this effect is related to our compound, showing its contribution to the barrier resistance of the stratum corneum.

To further elucidate the inflammatory landscape, we quantified M2 macrophages using the CD163 marker, a receptor highly expressed in cells responsible for resolving inflammation and promoting tissue repair.^48^ Our findings revealed a significant decrease in the percentage of CD163+ cells in the C+ group compared to all other groups. This suggests that MG suppresses M2 macrophage activity, thereby delaying inflammatory resolution and prolonging the tissue’s pathological state. These results corroborate our oxidative stress data, indicating that MG induces “OxInflammation”, a self-perpetuating feedback loop between oxidative stress and chronic inflammation that causes subclinical tissue damage.^49^

No significant increase in 8-hydroxy-2′-deoxyguanosine (8OHdG), a marker of oxidative DNA damage,^50^ was observed in any group, including the methylglyoxal-exposed group. This finding suggests that, under the experimental conditions employed, the oxidative insult may not have been sufficient to induce detectable nuclear DNA oxidation in the explants. Nevertheless, other oxidative stress markers evaluated in this study, including MDA, protein carbonylation, 4HNE, and antioxidant enzyme modulation, confirmed that oxidative stress was successfully established. Therefore, the oxidative damage induced by methylglyoxal may have preferentially affected lipids and proteins rather than DNA in this experimental model. Future studies including additional DNA damage markers may help further characterize the extent of genotoxic stress in human skin explants. In addition to DNA damage, we also analyzed 4-hydroxynonenal (4-HNE), a reactive byproduct of oxidative stress and lipid peroxidation, which has high reactivity with proteins, DNA, and other biomolecules.^51^ The byproducts of lipid peroxidation are more stable and react with target molecules, even if they are far away.^52^ In our results, the C+ group showed increased 4HNE levels compared to C–. Treatment with the nanoemulsion containing the active ingredient (N) showed no significant difference compared to C–, indicating attenuation of lipid peroxidation. Interestingly, the blank nanoemulsion (NS) exhibited even lower 4-HNE levels compared to both C– and N groups. These findings suggest that the nanoemulsion system itself may contribute to reducing oxidative damage and limiting lipid peroxidation reactions. Therefore, both the carrier system and the active compound may be involved in modulating oxidative stress responses observed in this study.^53^

Further studies are still required to better elucidate the mechanism of action of indole-2-carboxylic acid. Nevertheless, the results obtained in the present study demonstrated that the developed nanoemulsion exhibited significant antioxidant and anti-inflammatory activities, highlighting its potential as a promising candidate for the treatment of inflammatory skin disorders. Moreover, the nanoemulsion presented physicochemical characteristics comparable to several topical nanoemulsion systems previously described in the literature, particularly those employing nonionic surfactants, such as Tween and Span derivatives, for anti-inflammatory and transdermal delivery applications.^54^

## Conclusion

Taken together, these findings suggest that a nanoemulsion containing indole-2-carboxylic acid confers multifaceted protection against oxidative and inflammatory stress in skin, acting through redox homeostasis, structural preservation, and modulation of immune markers. These attributes support its potential use in dermocosmetic formulations designed for sensitive or inflamed skin, especially in contexts involving barrier disruption and oxidative stress.
